# Evaluating high-risk breast cancer surveillance outcomes in BRCA mutation carriers

**DOI:** 10.1007/s12282-026-01822-x

**Published:** 2026-02-03

**Authors:** Yuk-Kwan Chang, Wing-Pan Luk, Ling-Hiu Fung, Ava Kwong

**Affiliations:** 1 Department of Surgery, Queen Mary Hospital and Tung Wah Hospital, Hong Kong, Hong Kong S.A.R., China; 2 Research Department, Hong Kong Sanatorium and Hospital, Hong Kong, Hong Kong SAR, China; 3 Division of Breast Surgery, Department of Surgery, School of Clinical Medicine, University of Hong Kong, Hong Kong Hereditary Breast Cancer Family Registry, Breast Surgery Centre and Cancer Genetics Centre, Hong Kong Sanatorium and Hospital, Hong Kong, Hong Kong SAR, China

**Keywords:** BRCA mutation, Hereditary breast cancer, Surveillance, Asia, Risk-reducing mastectomy

## Abstract

**Background:**

Long-term outcome on breast cancer surveillance in BRCA mutation carriers are limited, especially in Asian populations where uptake of preventive surgery is low.

**Methods:**

We conducted a retrospective cohort study of 722 female *BRCA1* and *BRCA2* mutation carriers enrolled between January 2007 and December 2024 in the Hong Kong Hereditary Breast Cancer Family Registry. Of these, 702 (97.2%) underwent structured high-risk surveillance comprising biannual clinical breast examination and alternating 6-monthly breast magnetic resonance imaging and mammography or digital breast tomosynthesis with ultrasonography. The outcomes were breast cancer detection rate, stage, nodal status, interval cancer occurrence, and breast cancer–specific mortality.

**Results:**

Risk-reducing mastectomy was undertaken by 90 out of 722 carriers (12.5%). Surveillance detected 64 breast cancers during 4,398 imaging sessions (detection rate 1.5%). Most cancers (89.1%) were diagnosed at stage 0 or I, and 95.3% were node- negative. Interval cancers were uncommon. Among 304 carriers initially free of breast cancer who later developed the disease (*n* = 26), no breast cancer–specific death occurred. Overall, breast cancer–specific mortality in the cohort was 2.4%, with most deaths due to ovarian cancer with a median follow-up of 39.1 months (range, 6-212.3). Longer term follow-up is warranted.

**Conclusions:**

In this first Asian surveillance cohort of BRCA mutation carriers, prophylactic mastectomy uptake was low, yet structured screening enabled early-stage detection and minimal interval cancer. Surveillance should be considered as a key management strategy, with preventive surgery options individualized and cost-effectiveness evaluated.

## Background

Germline *BRCA1* and *BRCA2* pathogenic variants are associated with a significantly increased lifetime breast cancer risk of 50–85% [1], as well as elevated risks for ovarian, pancreatic, and prostate cancers [2, 3]. Among those with prior breast cancer, the risks of metachronous cancers are also substantially increased, especially in *BRCA1* mutation and those with young-onset disease [4, 5].

Breast cancer preventive strategies for BRCA mutation carriers include life-style modifications, surgery, chemoprevention, and enhanced surveillance. Risk-reducing surgery is the most effective and cost-efficient option compared to surveillance or chemoprevention [6]. A meta-analysis of 21 studies revealed that risk-reducing mastectomy (RRM) significantly reduces cancer risk by more than 90% [7], though the mortality benefit remains inconsistent [4, 8]. In an international study of more than 1600 women with BRCA pathogenic variants, RRM reduces the risk of breast cancer, and the probability of dying from breast cancer within 15 years after RRM was 0.95% [9] Recent studies suggest survival benefits in selected patients. A 2024 multicenter study of 5,290 young women (median age 35) with *BRCA1* and *BRCA2* mutations demonstrated that RRM improves overall survival, disease-free survival, and breast cancer-free interval [10]. Another study with 10-year follow-up showed significant survival benefits in *BRCA1* mutation carriers, but not in *BRCA2* [11]. RRM uptake in the West ranges from 27.8 to 50.0%, and is lower in Asia [12–14]. Bilateral salpingo-oophorectomy (BSO) effectively reduces ovarian cancer risk and improves survival, with higher global uptake (64.7%, range 36.4–76.3%) [14], but the benefits of breast cancer risk reduction remain controversial [15, 16]. Effective surveillance programs, even though not targeting tumor reduction but for early detection, remain essential.

While mammography (MMG) remains the standard screening tool for the general population, its sensitivity is lower in high-risk women, with interval cancers ranging from 29 to 50% and nodal involvement rates of 20–56% [17, 18]. This reduced performance is likely due to earlier cancer onset, higher breast density, and more aggressive tumor biology in BRCA mutation [19]. The use of digital breast tomosynthesis (DBT) instead of standard mammography alone might improve detection [20], but contrast-enhanced MRI offers superior sensitivity by assessing neovascularity and functional tissue characteristics [21]. A systematic review reported MMG sensitivity of 25–59% (pooled 39%) versus 68–100% for MRI (pooled 77%), with combined sensitivity reaching 94% [22]. MRI surveillance was also associated with reduced breast cancer mortality: in a cohort of 1,756 *BRCA1 and BRCA2* mutation carriers, 20-year mortality was 3.2% with MRI vs. 14.9% without, significantly lower in *BRCA1* carriers [23]. As a result, annual MRI from age 25–30 is now recommended in major guidelines for BRCA mutation management, including NCCN [24] and ESMO [25].

To optimize high-risk screening strategies for BRCA mutation carriers, various structured programs have been proposed in Western countries, aiming to detect cancers earlier and therefore reduce morbidity and potentially, mortality [19]. However, long-term outcome data remains limited. Furthermore, evidence on high-risk screening in Asian populations is scarce. Differences in genetic background [26], breast size and density [27, 28], and acceptance of risk-reducing surgery between Eastern and Western populations may influence outcomes. This study represents the first comprehensive analysis of a high-risk breast cancer surveillance program for female Chinese BRCA mutation carriers in Asia, evaluating cancer incidence, mortality, and the tumor stage upon cancer detection.

## Patients and method

This retrospective cohort study utilized data from a prospectively maintained database of the Hong Kong Hereditary Breast Cancer Family Registry. The registry includes individuals of Chinese ethnicity with confirmed germline pathogenic or likely pathogenic variants in breast cancer–related genes. Genetic testing was performed using a multigene panel (*BRCA1*,* BRCA2*,* TP53*,* PTEN*,* PALB2*, and *CDH1*) on peripheral blood samples, offered in accordance with local guidelines to patients with significant family history, early-onset disease, bilateral breast cancer, triple-negative breast cancer, or high-grade ovarian cancer [29]. 

Once a pathogenic variant was identified, cascade testing was offered to first-degree relatives aged 18 years or older after genetic counseling. Confirmed mutation carriers were managed in a dedicated high-risk clinic staffed by specialized clinicians and genetic counsellors. Management options included risk-reducing mastectomy (RRM) with or without immediate breast reconstruction (IBR), bilateral salpingo-oophorectomy (BSO), chemoprevention, or high-risk surveillance.

The surveillance protocol included biannual clinical breast examinations, annual mammogram (MMG) or digital breast tomosynthesis (DBT) with ultrasound (USG), and annual contrast-enhanced breast magnetic resonance imaging (MRI), scheduled alternately every 6 months. Suspicious findings were defined as BI-RADS category 4 or higher and reviewed by a multidisciplinary team comprising breast surgeons and radiologists. Further diagnostic workup, including additional imaging or biopsy, was performed as indicated. Upon breast cancer diagnosis, staging investigations were conducted when appropriate, and treatment options were discussed in multidisciplinary meetings. Surgical management included breast-conserving surgery or mastectomy with or without immediate breast reconstruction (IBR), with the option for concomitant prophylactic procedures (RRM, BSO).

Statistical analysis was performed with R software version 3.6.0. Continuous variables were displayed as median with range. Comparisons between categorical variables were performed with the Pearson chi-square test or the two-tailed Fisher’s exact test; *P* < = 0.05 was considered as statistically significant.

## Results

Between January 2007 and December 2024, 827 female *BRCA1* or *BRCA2* mutation carriers were managed in the registry. After excluding patients with terminal malignancies, those residing overseas, or who declined follow-up, 722 individuals without prior bilateral mastectomies were eligible for risk-reducing management. Of these, 20 (2.8%) underwent risk-reducing mastectomy (RRM) shortly following genetic diagnosis, while 70 patients (9.7%) later opted for RRM during surveillance, making the overall risk-reducing mastectomy uptake rate 12.5%. The median age of patients undergoing RRM was 47 years (range, 22–68), with 50% carrying *BRCA1* mutations and 76.7% having a prior history of breast cancer. The RRM uptake rate was higher in patients with breast cancer history: 14.7% (60 out of 408) compared with those without at 9.6% (30 of 314) (*P* = 0.0106).

BSO was recommended to mutation carriers after age 35–40 years old depending on mutation type and after completion of family per clinical guidelines. For *BRCA1* and those who were > = 35 years old, the uptake rate of BSO was 34.8% (64 out of 184 mutation carriers); while for *BRCA2* and those > = 40 years old, the uptake rate of BSO was 46.9% (106 out of 226 mutation carriers).

Among the 722 eligible individuals, 702 (97.2%) patients enrolled in the high-risk breast cancer surveillance program. Demographic and clinical characteristics of these 702 carriers are summarized in Table 1. *BRCA1* mutation carriers had a higher incidence of ovarian cancer compared with *BRCA2* carriers (32.6% vs. 14.5%). The median age at first cancer diagnosis was 42 years (range, 22–81) for breast cancer and 52 years (range, 17–84) for ovarian cancer.

**Table 1:** Demographics of *BRCA1* and *BRCA2* mutation carriers upon enrollment to high-risk surveillance (patient number = 702)

	*BRCA 1* (*n* = 337)	*BRCA 2* (*n* = 365)	Overall (*n* = 702)
Age of genetic testing (years), median (range)	48 (18–88)	47 (18–77)	47 (18–88)
*Age of cancer diagnosed (years), median (range)*			
Breast cancer	42 (22–81)	43 (22–74)	42 (22–81)
Ovarian cancer	51 (17–84)	56 (31–75)	52 (17–84)
*Personal history of cancer*,* no. (%)*			
Breast cancer only	147 (43.6%)	202 (55.3%)	349 (49.7%)
Breast and ovarian cancer	32 (9.5%)	17 (4.7%)	49 (7.0%)
Ovarian and/or other cancer	78 (23.1%)	36 (9.9%)	114 (16.2%)
Cancer-free, family of proband	80 (23.7%)	110 (30.1%)	190 (27.1%)
*Risk-reducing strategies adopted*,* no. (%)*			
^a^Risk-reducing BSO	64 (34.8%, out of 184)	106 (46.9%, out of 226)	170 (41.5%, out of 410)
Chemoprevention	4 (1.2%)	22 (6.0%)	26 (3.6%)

^a^Uptake rates of risk-reducing BSO were calculated after excluding those who received BSO for malignancy or other pathology before genetic testing

*BSO* Bilateral salpingo-oophorectomy

At the time of *BRCA* mutation detection and entry into surveillance, 49.7% had a prior history of breast cancer, 7.0% had both breast and ovarian cancers, 16.2% had a history of ovarian or other cancers, and 27.1% were cancer-free carriers identified through family testing.

Among the 702 patients in the enhanced surveillance program, a total of 4,398 breast imaging sessions were performed over 17 years, including standard mammography (MMG) or digital breast tomosynthesis (DBT) with ultrasound (USG), and breast MRI with contrast. Since 2014, all standard MMG were replaced by DBT in our radiology units. Sixty-four breast cancers were detected, yielding an overall cancer detection rate of 1.5% per screening session (15 per 1,000 screens).

Results of screening imaging modalities are summarized in Table 2. Suspicious findings occurred in 7.0% of MMG/DBT and 8.0% of USG sessions, while MRI yielded a higher rate of suspicious findings (13.1%). MRI demonstrated a higher cancer detection rate (1.7%) compared with MMG/DBT and USG (1.1%–1.2%), although the difference was not statistically significant (*P* = 0.234). Regarding the stage of tumor detection, MRI identified all cancers at Stage 0 or Stage 1. In comparison, MMG/ DBT and USG detected over 80% of cancers at Stage 0 or Stage 1. The rate of node-negative disease ranged from 88.5% to 100%, depending on the imaging modality.

**Table 2:** Results of screening sessions (patient number = 702)

	^a^MMG/DBT	^a^USG	MRI
Total number of imaging performed	2111	2316	2000
Number of breast imaging performed for each patient, median (range)	3 (1–14)	3 (1–15)	3 (1–13)
Number resulted in suspicious findings, no (%)	157 (7%)	179 (8%)	267 (13%)
^b^Number resulted in additional imaging	40 (1.9%)	48 (2.1%)	261 (13.1%)
^b^Number resulted in FNAC	14 (0.7%)	25 (1.1%)	16 (0.8%)
^b^Number resulted in core biopsy	80 (3.8%)	87 (3.8%)	104 (5.2%)
^b^Number resulted in VABB	7 (0.3%)	9 (0.4%)	16 (0.8%)
^b^Number resulted in excision	12 (0.6%)	11 (0.5%)	23 (1.2%)
^c^New cancer detected per screening, no (%)	25 (1.2%)	26 (1.1%)	33 (1.7%)
*TNM stage, no. (%)*			
Stage 0	10 (40.0%)	8 (30.8%)	13 (39.4%)
Stage I	11 (44.0%)	12 (46.2%)	19 (57.6%)
Stage II	3 (12.0%)	5 (19.2%)	0 (0.0%)
Stage III	1 (4/0%)	1 (3.8%)	0 (0.0%)
*T-stage, no. (%)*			
Tis	10 (40.0%)	8 (30.8%)	13 (39.4%)
T1	11 (44.0%)	13 (50.0%)	19 (57.6%)
T2	4 (16.0%)	5 (19.2%)	0 (0.0%)
*N-stage, no. (%)*			
N0	23 (92.0%)	23 (88.5%)	33 (100.0%)
N1	1 (4.0%)	2 (7.7%)	0 (0.0%)
N2	1 (4.0%)	1 (3.8%)	0 (0.0%)

*MMG* mammogram, *DBT* digital breast tomosynthesis, *USG* ultrasound, *MRI* magnetic resonance imaging, *FNAC* fine needle aspiration, *VABB* vacuum-assisted breast biopsy

^a^Screening session overlaps existed between MMG/DBT and USG, since MMG/DBT and USG were arranged to be performed in the same session

^b^Follow-up management from a suspicious screening session could have overlaps

^c^New cancers detected on surveillance across different imaging modalities could have overlaps

Table 3 summarizes the characteristics of 66 breast cancers diagnosed during the surveillance period. Of these, 64 were detected through surveillance imaging, and 2 were ductal carcinoma in situ (DCIS) incidentally identified on risk-reducing mastectomy specimen. The median age at diagnosis was 48.5 years (range, 33–73).

**Table 3:** Characteristics of the newly diagnosed breast cancer during surveillance period

		^a^Total (*n* = 66)	*BRCA1* (*n* = 35)	*BRCA2* (*n* = 31)	*P*-value
Age at diagnosis of new cancer (median; range)		48.5 (33–73)	48 (33–72)	50 (34–73)	0.995
No. of screening imaging from surveillance to breast cancer diagnosis (median; range)		4 (1–20)	4 (1–17)	3 (1–20)	0.446
BSO already done, no (%)		21 (31.8%)	12 (34.3%)	9 (29.0%)	0.792
TNM stage, No (%)	Stage 0	27 (40.9%)	12 (32.3%)	15 (48.4%)	0.356
	Stage I	33 (50.8%)	20 (58.8%)	13 (41.9%)	NA
	Stage II	5 (7.7%)	3 (8.8%)	2 (6.5%)	NA
	Stage III	1 (1.5%)	0 (0.0%)	1 (3.2%)	NA
T-stage, No (%)	Tis	27 (40.9%)	12 (32.3%)	15 (48.4%)	0.285
	T1	34 (52.3%)	21 (61.8%)	13 (41.9%)	NA
	T2	5 (7.7%)	2 (5.9%)	3 (9.7%)	NA
N-stage, No (%)	N0	63 (95.4%)	34 (97.1%)	29 (93.5%)	0.735
	N1	2 (3.1%)	1 (2.9%)	1 (3.2%)	NA
	N2	1 (1.5%)	0 (0.0%)	1 (3.2%)	NA
IHC profile, No (%)	ER/PR + HER2-	17 (43.6%)	5 (20.8%)	12 (80%)	< 0.001
	ER/PR + HER2 +	3 (7.7%)	2 (8.3%)	1 (6.7%)	NA
	ER/PR - HER2 +	0 (0%)	0 (0.0%)	0 (0.0%)	NA
	ER/PR - HER2 -	17 (43.6%)	16 (66.7%)	1 (6.7%)	NA
	ER/PR + HER2 Equivocal	2 (5.1%)	1 (4.2%)	1 (6.7%)	NA

*BSO* bilateral salpingo-oophorectomy, *n* number, *is* in situ, *IHC* immunohistochemistry

^a^Including 64 breast cancers diagnosed by surveillance breast imaging and 2 breast cancers (ductal carcinoma in situ) incidentally found after risk-reducing mastectomy

Excluding the incidental DCIS cases, 89.1% of cancers were diagnosed at stage 0 (*n* = 24) or stage I (*n* = 33), with a node-negative rate of 95.3% (*n* = 61). Two patients were diagnosed with cT2N2 and cT1N1 disease within 3 months of genetic testing and were presumed to have preexisting undetected cancers prior to registry enrollment. Another patient with cT2N1 disease missed two consecutive surveillance sessions. No other interval cancers were identified during the 6-month screening intervals. Among invasive cancers, 43.6% were triple-negative, all others were estrogen receptor (ER) positive. In *BRCA1* mutation carriers, 66.7% of cancers were triple-negative.

Management details for the 64 patients with surveillance-detected breast cancer are summarized in Table 4. One patient declined surgery and sought alternative treatment. Among the remaining, 25.4% (*n* = 16) underwent breast-conserving surgery, 52.4% (*n* = 33) had mastectomy alone, and 22.2% (*N* = 14) underwent mastectomy with immediate breast reconstruction. Axillary lymph node dissection was performed in 4.8% (*n* = 3) of cases due to confirmed nodal metastasis.

**Table 4:** Management of the newly detected breast cancer under surveillance program (^a^number = 63)

	No. (%)
*Cancer-side surgery*	
Breast conservation	16 (25.4%)
Mastectomy only	33 (52.4%)
Mastectomy + reconstruction	14 (22.2%)
Axillary dissection	3 (4.8%)
*Risk-reducing surgery*	
^b^ RR mastectomy (in 39 patients with contralateral breast)	20 (51.3%)
^c^ RR BSO (in 58 patients with intact ovaries)	28 (48.3%)
*Neoadjuvant/adjuvant therapy*	
Neoadjuvant systemic therapy	3 (4.8%)
Adjuvant systemic chemotherapy	21 (33.3%)
Adjuvant radiotherapy	16 (25.4%)

*RR* risk-reducing,* BSO* bilateral salpingo-oophorectomy

^a^Excluded 2 patients whose breast cancer was found incidentally after risk-reducing mastectomy and 1 patient who refused surgery and treatment

^b^Percentage calculations excluded those who already had contralateral mastectomy

^c^Percentage calculations excluded those who already had BSO

Of those diagnosed through surveillance, 51.3% (*N* = 20) chose to undergo contralateral risk-reducing mastectomy. Although most cancers were detected at an early stage, 3 patients (cT2N2, cT2N1, and cT1N1) received neoadjuvant systemic therapy, due to nodal positive status and triple negative or luminal B biology. Adjuvant chemotherapy was administered in 33.3% (*n* = 21), and 11 (*n* = 16) patients required adjuvant radiotherapy due to breast-conserving surgery or nodal involvement.

Among these 64 surveillance-detected breast cancers, 20.3% (*n* = 13) occurred in patients aged ≤ 40 years. Within these young onset patients, 61.5% (*n* = 8) were *BRCA1* mutation carriers, 84.6% (*n* = 11) were diagnosed at stage 0 or I. and 69.2% (*n* = 9) cases were triple negative. The mastectomy rate in this younger cohort was 75.0%, similar to that of the older patients; however, the rate of immediate reconstruction was higher at 50.0%. Additionally, 41.7% underwent contralateral risk-reducing mastectomy during the same operation.

Table 5 summarized the surveillance outcomes among the 702 patients. The median follow-up duration was 39.1 months (range, 6–212.3). A total of 78 breast cancer events were identified: 66 were new primary cancers, as previously described, and 12 were local recurrences, defined as ipsilateral tumors with same immunohistochemical profiles and, for prior breast-conserving surgery, recurrence in the same quadrant. The overall breast cancer occurrence rate was 11.1% (78 out of 702). Among 163 patients with a history of ovarian and/or other cancers, the new breast cancer incidence was 5.3% (6 of 114 patients).

**Table 5:** Outcome for mutation carriers according to disease status on presentation (patient number = 702)

Outcome, no. (%)	Breast cancer (*n* = 349)	Breast and ovarian cancer (*n* = 49)	^a^ Ovarian cancer and/or other cancer (*n* = 114)	Cancer-free (*n* = 190)	Overall (*n* = 702)
Cancer-free/in remission	281 (80.5%)	32 (65.3%)	90 (78.9%)	168 (88.4%)	571 (81.3%)
Breast cancer occurrence	48 (13.8%)	4 (8.2%)	6 (5.3%)	20 (10.5%)	78 (11.1%)
Breast cancer-specific mortality	14 (4.0%)	3 (6.1%)	0 (0.0%)	0 (0.0%)	17 (2.4%)
Other cause mortality	6 (1.7%)	10 (20.4%)	18 (15.8%)	2 (1.1%)	36 (5.1%)

^a^109 carriers with ovarian cancer history and 5 carriers with other cancer history

Breast cancer–specific mortality was 2.4% (17 of 702), occurring exclusively in patients with a prior history of breast cancer (17 of 398; 4.3%). Notably, among the 26 patients who developed breast cancer during surveillance without prior history, no breast cancer–specific deaths were observed.

Other-cause mortality occurred in 5.1% (*n* = 36), of which 83.3% (*n* = 30) were attributed to recurrence or progression of ovarian cancer. Mortality was highest in patients with prior ovarian cancer: 27.1% in those with both breast and ovarian cancer and 15.8% in those with ovarian and/or other cancers.

## Discussion

Internationally, the uptake of RRM among BRCA mutation carriers ranges from 27.8% to 50.0% [12, 13]. Data from Asian populations are limited. A study from Singapore reported RRM rates of 0.46%–1.25% among high-risk women, but this data was not specific to BRCA mutation carriers [30]. In our cohort, RRM was discussed at multiple time points—after genetic diagnosis, during surveillance, and at the time of breast cancer diagnosis. After excluding patients with prior bilateral mastectomy, the overall RRM uptake was 12.5%, it was particularly low among those without prior breast cancer at a rate of 9.6%. Conversely, breast cancer surveillance was accepted by over 97% of eligible carriers, highlighting a preference for a conservative approach in our population.

High-risk surveillance remains the core management for *BRCA1* and *BRCA2* mutation carriers in our cohort. In this study, the breast cancer detection rate was 1.5% per screening session, with 89.1% diagnosed at stage 0 or I and a 95.3% node-negative rate. Interval cancers between 6-month screenings were minimal. These results are also comparable, if not favorable, to international high-risk surveillance programs. Laitman et al. reported a detection rate of 1.2% (79 cancers from 6641 imaging studies) in 1055 BRCA mutation carriers, with 26.6% diagnosed as carcinoma in situ and 60.8% at stage I or II [31]. Bernstein-Molho et al. reported that 19.8% were diagnosed with carcinoma in situ and 56.3% had T1N0 disease in a cohort of 298 BRCA mutation carriers [32].

Despite early-stage diagnosis in most of our patients, approximately one-third required chemotherapy. This might be due to a high proportion of young onset and triple-negative breast cancer. Among patients with breast cancer diagnosed ≤ 40 years old during surveillance (*n* = 13, 20.3%), over 60% were *BRCA1* mutation carriers, and almost 70% had triple-negative tumors. Recently, an international cohort study with more than 5000 BRCA mutation carriers has provided robust evidence that RRM was associated with a significant improvement in overall survival in patient with young age of onset of breast cancer < = 40 years old [10]. Prior cohort studies also suggested that risk-reducing mastectomy may confer survival benefits in *BRCA1* mutation [11] and contralateral risk-reducing mastectomy could improve survival [33]. For survival benefits, and avoidance of cancer occurrence and morbidities from oncological treatment, risk-reducing mastectomies should be adequately discussed during counseling sessions for selected individuals with young age of onset, and particularly *BRCA1* mutation carriers.

Breast cancer incidence during surveillance varied by patients’ cancer history. A lower rate of breast cancer occurrence was observed in those with a prior history of ovarian cancer (5.3% in a median follow-up of 39 months). This observation aligns with findings from other cohorts [34, 35]. In a study of 192 *BRCA*-positive patients with a history of epithelial ovarian cancer (EOC), 16 (8.3%) developed breast cancer at a median of 50 months (range, 5–327) after EOC diagnosis [34]. Another study showed 10% out of 184 patients with *BRCA*-associated EOC were diagnosed with breast cancer at a median of 48 months following EOC [36]. Whether BSO reduces breast cancer risk remains controversial; *BRCA* mutation carriers after BSO or treatment for EOC should adhere to breast cancer surveillance program. However, given the overall survival in this cohort was primarily driven by ovarian cancer-related mortality, deprioritizing risk-reducing mastectomy in patient with prior EOC could be appropriate, especially considering the prognosis and stage of EOC.

This study has several limitations. First, it could not directly evaluate whether high-risk surveillance reduces breast cancer–specific mortality in BRCA mutation carriers, as all participants were enrolled in surveillance without a non-surveillance comparator. Potential mortality reduction can only be inferred from the high proportion of early-stage diagnoses, although lead-time bias cannot be excluded. Second, the median follow-up duration of 39.1 months is too short for assessing long-term survival outcomes, such as mortality in hereditary breast cancer, which could appear after 10–20 years. Further long-term follow up is necessary, in order to analyze overall survival, disease-free survival and contralateral cancer risk. Third, the study did not demonstrate superior sensitivity of MRI compared with DBT and USG, which contrasts with prior multicenter data showing MRI outperforms other modalities in high-risk populations [19], and could not determine the surveillance performance for each modality and their different combinations. However, this study was not designed to compare imaging modalities; rather, it evaluated a structured surveillance protocol that included alternating MRI and combined DBT/USG at 6-month intervals. Overlaps in cancer detection between modalities were not examined and may have influenced detection attribution. Fourth, selection bias could be present since those who enrolled into the surveillance program would have relatively good premorbid condition and may bias towards better survival outcomes.

In conclusion, the uptake of RRM among *BRCA* mutation carriers in our cohort was low (12.5%), whereas participation in high-risk breast cancer surveillance exceeded 97%. Our surveillance protocol—a first to report internationally with alternating MRI and DBT with USG at 6-month intervals and a first to report high risk surveillance outcome in Asia—yielded a cancer detection rate of 1.5% per screening, with 89.1% diagnosed at stage 0/I, 95.3% node-negative, and minimal interval cancers. Overall mortality was primarily driven by ovarian cancer history in a median follow-up of 39 months. Risk-reducing strategies, including RRM, should be individualized based on patient-specific factors such as age at diagnosis, mutation type, and ovarian cancer history and prognosis. Further studies, including cost-effectiveness analyses, are warranted to inform public health planning and optimize management strategies for high-risk populations.

## Data Availability

Data is available from the corresponding author upon reasonable request.
